# Corrosion and Ion Release in 304L Stainless Steel Biomedical Stylets

**DOI:** 10.3390/ma18163769

**Published:** 2025-08-11

**Authors:** Lucien Reclaru, Alexandru Florian Grecu, Daniela Florentina Grecu, Cristian Virgil Lungulescu, Dan Cristian Grecu

**Affiliations:** 1 Scientific Independent Consultant Biomaterials and Medical Devices, 103 Paul-Vouga, 2074 Marin-Epargnier, Switzerland; lreclaru@gmail.com; 2 Department of Orthopedics and Traumatology, University of Medicine and Pharmacy of Craiova, 540053 Craiova, Romania; dan.grecu@umfcv.ro; 3 Filantropia Municipal Clinical Hospital of Craiova, 540053 Craiova, Romania; 4 Department of Oncology of Craiova, University of Medicine and Pharmacy of Craiova, 540053 Craiova, Romania; cristian.lungulescu@umfcv.ro

**Keywords:** austenitic steels 304 L (DIN 1.4306) uniform corrosion, pitting corrosion, crevice corrosion, intergranular corrosion, cations release artificial sweat, plasma bone stylet medical devices

## Abstract

Styles are invasive medical devices that are visible on images and are used in several medical specialties, including cardiology, neurology, orthopaedics, anaesthesia, oto-rhino-laryngology (ENT), and dentistry. With their thin and flexible design, they allow for the optimal positioning and precise guidance of medical devices such as nerve stimulation, defibrillation, electrode positioning, and catheter insertion. Generally, they are made of stainless steel, offering a combination of rigidity and flexibility. The aim of this study is to evaluate the sensitivity of austenitic stainless steel 304L used for the manufacture of J-stylets in uniform, pitting, crevice, and intergranular corrosion. We follow the manufacturing process step by step in order to analyse the risks of corrosion sensitisation and the cumulative effects of various forms of degradation, which could lead to a significant release of metal cations. Another objective of this study is to determine the optimal heat treatment temperature to minimise sensitivity to the intergranular corrosion of 304L steel. Uniform corrosion: Two samples were taken at each stage of the manufacturing process (eight steps in total), in the form of rods. After one hour of immersion, potentiodynamic polarisation curves were plotted up to ±400 mV vs. SCE. A coulometric analysis was also performed by integrating the anode zone between E (i = 0) and +400 mV vs. SCE. The values obtained by integration are expressed as mC/cm^2^. The test medium used was a simulated artificial plasma solution (9 g/L NaCl solution). Intergranular corrosion: (a) Chemical test: Thirty rod-shaped samples were tested, representing the eight manufacturing steps, as well as heat treatments at 500 °C, 620 °C, and 750 °C, in accordance with ASTM A262 (F method). (b) Electrochemical Potentiokinetic Reactivation (EPR) according to ASTM G108–94 (2015). Two samples were tested for each condition: without heat treatment and after treatments at 500 °C, 620 °C, and 750 °C. Release of cations: The release of metal ions was evaluated in the following two media: artificial sweat, according to EN 1811:2011+A1:2015, and bone plasma, according to the Fitton-Jackson and Burks-Peck method. Six samples that had been heat-treated at 500 °C for one hour were analysed. Results, discussions: (a) Analysis of the polarisation curves revealed significant disturbances in the heat treatment steps, as well as the μC/cm^2^ quantities, which were between 150,000 and 400,000 compared to only 40–180 for the other manufacturing steps; (b) Electrochemical Potentiokinetic reactivation (EPR) tests indicated that the temperature of 500 °C was a good choice to limit 304L steel sensitisation in intergranular corrosion; and (c) the quantities of cations released in EN 1811 sweat were of the order of a few μg/cm^2^ week, as for Fe: 2.31, Cr: 0.05, and Ni: 0.12.

## 1. Introduction

In the medical field, precision is an important factor during procedures such as stimulation, defibrillation, and complex neurological interventions. The stylet is a device used in these practices. It plays a very important role in ensuring the optimal positioning and precise guidance of medical devices.

- *In cardiac stimulation*, the stylet optimises the placement of the electrodes of implantable pacemakers and defibrillators, thus ensuring the perfect transmission of electrical impulses. Its use is inevitable to avoid complications and ensure effective therapy. Stylets are also used in cardiac catheterisation procedures to guide angiography or angioplasty intervention catheters. They allow for positioning of the electrodes to ensure optimal stimulation [1,2,3,4,5,6,7,8,9,10,11].
- *In neurology*, procedures such as deep brain stimulation and targeted drug administration require control of instruments. The stylet is used to provide accurate control of a device, reduce the risk of errors, and improve clinical outcomes [12,13,14,15,16].
- *In orthopaedics*, stylets are mainly used for implant guidance: they help to precisely position screws, pins, and intramedullary rods. Thus, they grant minimally invasive access and allow for creating a passage for inserting devices without damaging the surrounding tissues. They also allow for surgical navigation associated with imaging systems such as radiography, fluoroscopy or a puncture, and biopsy. Some styli are used to collect bone fragments [17,18,19,20,21].
- *In oto-rhino-laryngology* (*ENT*), stylets are used to guide or support other devices during diagnostic or surgical procedures. For example, they are used in difficult naso-tracheal or orotracheal intubation, middle ear surgery (tympanoplasty, ossiculoplasty), nasal endoscopic or sinus and laryngoscopy surgery, and vocal cord interventions [22,23,24,25,26,27,28,29,30,31,32,33,34,35].
- *In anaesthesia and pain management*, they are used for the insertion of needles into regional nerve blocks and epidural anaesthesia. They allow for precise positioning of epidural or spinal catheters [36,37].

The following are examples of stylets used in dental applications:

- *Endodontics* for the treatment of root canals. They are used to locate and block root canals before mechanical instrumentation. They also allow for navigation in narrow and curved channels, often in addition to endodontic files [38].
- *In apical surgery/microsurgery*. They are used to guide very fine instruments or micro-tools in hard-to-reach areas, particularly during apical resection (amputation of the root end) [39].
- *In implantology*. In some implant guidance techniques, a very thin metal stylus can be used to check the alignment, direction, or depth of the bone drill. They can also help to test bone density before implant insertion [40].
- *In periodontal/minimally invasive surgery*. Some surgical stylets allow for gentle tissue dissection or the delineation of areas to be treated, particularly in conservative approaches [40,41,42].
- *Paediatric or diagnostic*. Very fine exploratory styli are used to detect micro-fractures, incipient caries, or structural abnormalities [43,44].

Innovation in stylus design, with advanced materials and sophisticated guiding technologies, allows for excellent levels of precision to be achieved today. These advances contribute directly to improved patient care and the success of critical medical interventions [45].

Their fine and flexible design allows for precise and controlled insertion into delicate structures of the human body. Their fine and flexible tip suggests applications in procedures requiring delicate guidance, such as nerve stimulation, defibrillation, and the insertion of catheters.

*Structure and design:* Their tapered end is rounded to minimise tissue trauma, and some models have markings for precise positioning under fluoroscopy or medical imaging. They are designed with different sizes and curves adapted to the specific needs of each intervention.

The materials used in their manufacture are usually stainless steel, types 304 L DIN 1.4306, DIN 1.4307) and 316L (DIM 1.4435, DIN 1.4441), offering a combination of rigidity and flexibility. They are sometimes coated with polymers to reduce friction and improve biocompatibility.

Since they are single-use stylets, considering material and manufacturing costs, they are usually made of steel type 304L (DIN 1.4306).

Type 304L is an austenitic stainless steel with a medium corrosion resistance, intermediate between that of 302 and 316. Its limited carbon content specifically prevents intergranular corrosion, but it does not contain molybdenum, which improves resistance to non-oxidising acids and pitting and crevice corrosion. It shows limited machinability, so complex machining is preferred to the use of austenitic steels from the family 316L.

The manufacture of stylets involves a series of precise and technically demanding operations, including rectifying, wire drawing, bevelling, rounding, and tapering, as well as cold forming, electrolytic or mechanical polishing, heat treatment, ultra-cleaning, passivation, and sterilisation.

Austenitic-type stainless steels show weaknesses in localised forms of pitting, crevice, and intergranular corrosion.

The electrical potentials needed to trigger these morphologies can be determined from potentiodynamic scanning polarisation curves or chemical wave attacks.

The factors that can generate these forms of corrosion are usually a seed joint attack, stress process, surface depassivation, or other processes. Thus, we investigated the phases of fabrication step by step, ending in the manufacture of the stylet.

The corrosion process is dependent on a multitude of factors, such as physico-chemical factors (chemical composition and microstructure of the alloy, temperature, pH, chemical composition of the environment, etc.) and mechanical factors (stress, friction, etc.) [46]. The relationship between corrosion rate and grain size has been demonstrated in many studies [47,48,49]. Its importance lies in the fact that this parameter can be adapted by producers [47,48,49,50]. In general, the composition of austenitic stainless steels is adapted to meet service requirements in various corrosive environments [47].

Another aspect of particular interest in our study is the quantity of released cations.

Allergenic, toxic/cytotoxic, mutagenic, or carcinogenic (e.g., Ni, Co, Cr, V, and Al) species may be released into the body during corrosion processes.

The role of nickel in the biological response to alloys used in medical devices is of immense importance regarding toxicology and biological performance. However, this requires careful evaluation, as no compromise is acceptable with respect to mechanical properties, corrosion resistance, or any other possible adverse consequences due to nickel substitution [51,52].

Another aspect of austenitic steels is the release of nickel on contact with the skin. Nickel allergy is the most common of all contact allergies. In the European population, the prevalence of nickel allergy is 10–15% among adult women and 1–3% among adult men [53,54,55,56,57]. In total, 30% of nickel-sensitive people in the general population develop hand eczema. Adolescents and young adults tend to have a higher prevalence due to frequent piercings.

In Europe, for objects containing nickel intended for permanent contact with the skin, imposed a ban if the rate of nickel release exceeds 0.5 µg/cm^2^.week. The subsequent “Piercing in the Human Body” specifies that the limit rate of nickel release for these cases is 0.2 µg/cm^2^.week [58,59].

Corrosion resistance is an essential criterion in the selection of materials for biomedical applications. Indeed, the degradation of materials through corrosion not only entails high economic costs, but also significant environmental and biological consequences, especially during the contact time between biomaterials and living tissues [60,61,62]. Stainless steels, although appreciated for their relative inertia, may be subject to chemical corrosion, which can cause the release of toxic metal ions into surrounding tissues. This progressive degradation can also compromise the intrinsic physico-mechanical properties of the material over time [60,61].

The aim of this study is to evaluate the sensitivity of austenitic stainless steel 304L to the following different forms of corrosion: uniform, pitting, crevice, and intergranular. The main objective is to follow the manufacturing process of a stylus step by step, in order to analyse the sensitisation risks and the cumulative effects of various forms of degradation, likely to lead to a significant release of metal cations. Such release could compromise the biocompatibility of the device, making it unacceptable for clinical use.

Another objective of this study is to determine the optimal heat treatment temperature to minimise the intergranular corrosion sensitisation of 304L steel.

The expected results should be closely correlated with the key stages of the manufacturing process: straightening, stretching, shaping (bevelled, rounded/bulbed, and tapered), cutting to length, polishing or electropolishing, etc.

The novelty of this work lies in an integrated approach that connects, in a scientific manner, structural sensitisation by thermal treatment, electrochemical behaviour, the cleaning and passivation of surfaces, and the different stages of the industrial process for manufacturing medical devices with 304L. This study provides concrete elements to optimise manufacturing parameters in order to ensure long-lasting corrosion resistance and clinical safety.

## 2. Materials and Methods

Table 1 shows the composition of the austenitic stainless steels which were used to prepare the samples for the corrosion evaluation tests [63,64].

**Figure 1:**
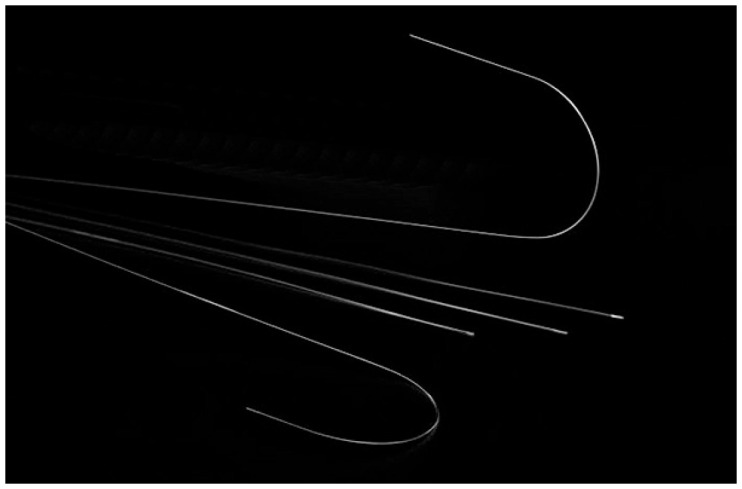
Stylets dispositive—medical.

The image in Figure 1 shows a medical stylus used for specific procedures in cardiology, neurology, and anaesthesia. Its fine and flexible tip suggests application in procedures requiring delicate guidance, such as nerve stimulation, defibrillation, and the insertion of catheters. Different sizes and curvatures can be adapted to the specific needs of each surgical procedure.

**Figure 2:**
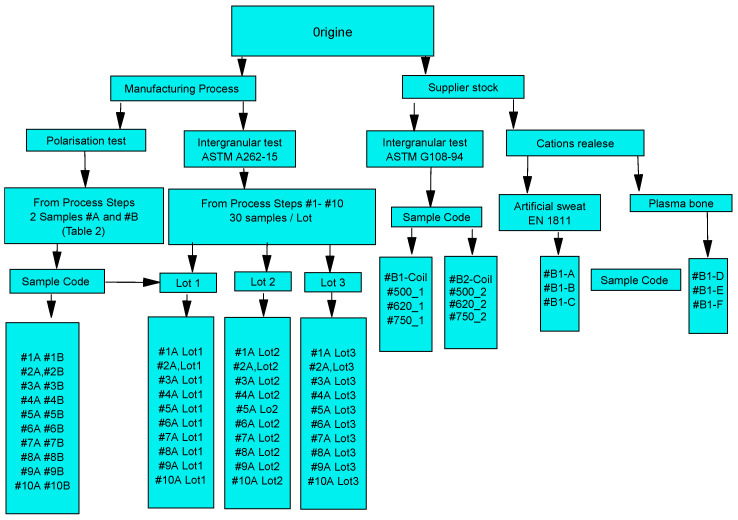
Diagram: Identification of samples used in the tests.

For a better understanding, we built a flow chart of the research, in which the tests carried out, the origin of the samples, and the number of samples used for each measurement technique are presented (Figure 2). We also indicate the codes of the samples used in the evaluation tests. The objective is to allow the reader to better orient themselves in reading this work.

### 2.1. Sample Designation

**Figure 3:**
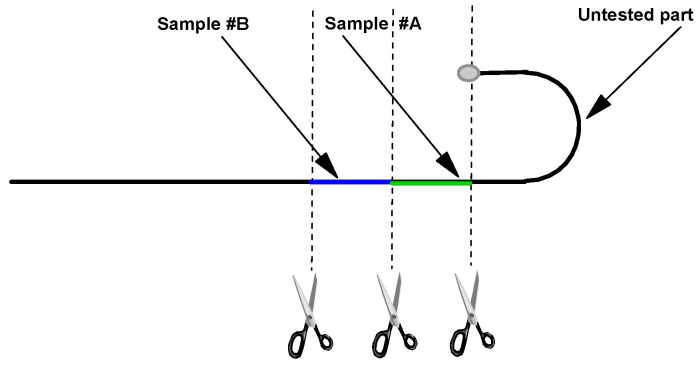
Sample preparation.

Samples from wire stylets for electrochemical corrosion evaluation were prepared specifically for each investigation. The samples were in the form of rods 25 mm long and Ø 0.360 mm; they were cut from the Wire Stylet J side of the stylus (Figure 3)

The series of samples tested were grouped as follows (Table 2)

### 2.2. Manufacturing Process Steps for a Medical Wire Stylet

The operating range according to Table 2 is very complex and requires very strict control for each manufacturing step.

1. The choice of material is crucial and depends on the required flexibility, strength, and biocompatibility. In general, stainless steels of grades 304, 304L, and 316L are used.
2. This is an operation used to correct the deformation or curvature of metal materials (wires, bars, tubes, etc.) in order to make them straight and improve their dimensional and mechanical characteristics.
3. The straightened raw metal rod is pulled through a series of dies to reduce its diameter, improve the surface finish, and ensure the narrow dimensional tolerances of the wire.
4. Degreasing of the wire with non-chlorinated solvents and alcohol-like products.
5. Depending on the application, the stylet tips may be bevelled, rounded, or tapered to enhance ease of insertion and minimise tissue trauma.
6. Depending on the application, the distal tip may be formed into a straight, J-shape, or spiral profile. Specific geometries enhance navigability and reduce trauma during insertion.
7. Wires are annealed to relieve internal stresses, achieve the desired mechanical properties, and create variable flexibility along the wire length.
8. Heat treatment at 750 °C.

### 2.3. Corrosion Assessment by Electrochemical Techniques

**Figure 4:**
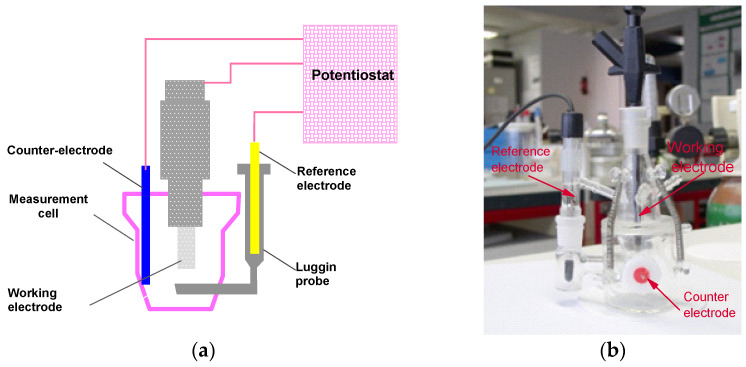
Electrochemical assembly. (**a**) Three-electrode measurement and (**b**) electrochemical cell.

Electrochemical measurements were made with a potentiostatic assembly of the following three electrodes: one working electrode, one platinum counter electrode, and one ECS (saturated calomel) reference electrode. The measurement system was operated by an EG&G Par 273A potentiostat modified with 1 pA background noise. A schematic representation of the technique used is shown in Figure 4a. The corrosion cell was a glass cell (Figure 4b). The measurement system was protected by a Faraday cage.

The test milieu is an artificial plasma solution (NaCl 9 g/L). For each measurement, 2 mL of electrolyte is used. The sample area is 0.226 cm^2^. For the evaluation of sensitivity to generalised corrosion, the electrochemical quantities measured are as follows:

- Immersion in the electrolyte for 1 h with potential recording in open circuit.
- Recording of polarisation potentidynamic curves (±400 mV vs. SCE).
- Coulometric analysis in the anodic zone in the range E (i = 0) at +400 mV ECS.

### 2.4. Evaluation of Intergranular Corrosion Sensitisation

- Chemical test

Various tests to evaluate the sensitivity of stainless steels to intergranular corrosion have been described by several authors [65,66,67]. The methods recommended by ASTM A262-15 [68] are summarised in Table 3, which we used for our tests.

We evaluated the sensitivity of 304L steel according to Practice E in Table 4. The samples were rods with a length of 25 mm taken from stylets from three batches. From each batch, we took only sample #A. We tested 3 samples by step manufacturing (10 steps) for three lots (coils 1–3), totalling 90 samples.

**Figure 5:**
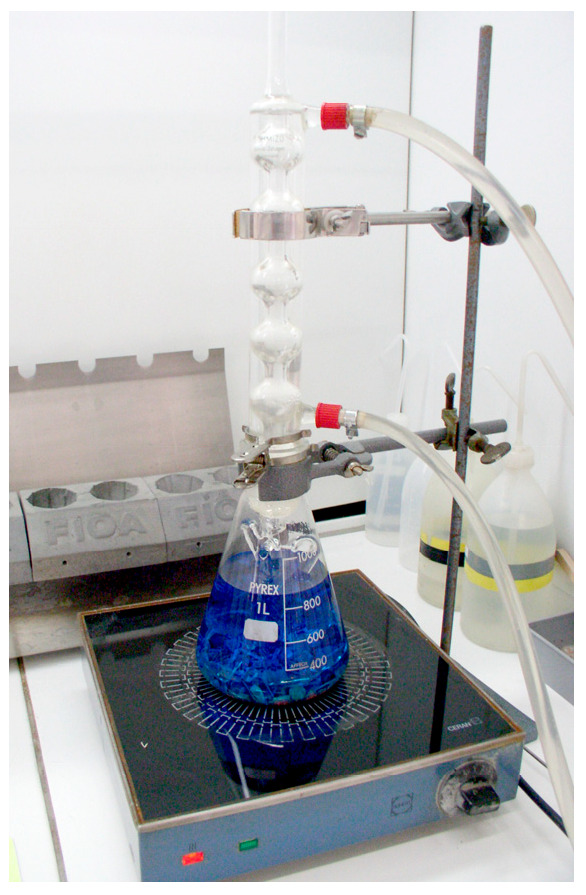
Equipment according to ASTM A262.

**Figure 6:**
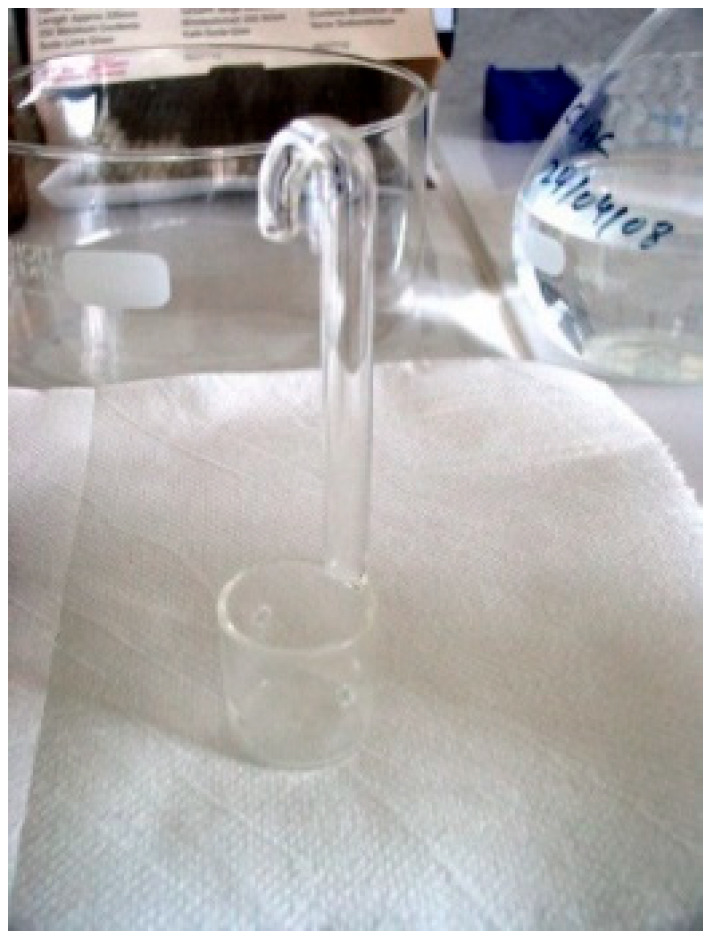
Cradle for the metal sample to be tested.

The test setup was ASTM A262-15 [68] (Figure 5). The samples to be tested were placed in a specific glass cradle, as shown in Figure 6. Mass loss using a micro balance was determined after testing for each sample. An optic or SEM examination was also performed for each.

- Electrochemical tests

EPR (*Electrochemical* Polarization Potentiodynamic Reactivation) measurements, single or double loop, are methods for examining and assessing the corrosion sensitivity of austenitic steels [36,69,70,71,72,73,74,75,76,77].

ASTM G108-94 (2015) [77], practice E (see Table 3), uses the single-loop technique to assess susceptibility to intergranular corrosion. In this test, a sample previously polished to a 1 μm finish is polarised for two minutes at +200 mV with respect to the saturated calomel electrode (SCE), in a solution containing 0.5 M H_2_SO_4_ and 0.01 M KSCN. Then, the potential is decreased at a constant rate of 6 V/h to the corrosion potential (Ecorr). This potential decay causes the reactivation of the sample, leading to the breakdown of the passive film in chromium-depleted areas.

**Figure 7:**
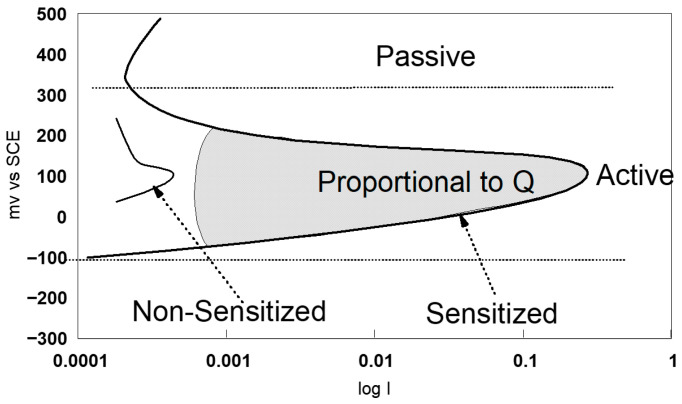
Procedures of single-loop EPR test method according to ASTM G108–94 (2015) [77].

The area under the loop generated on the current–potential curve (see Figure 7) corresponds to the electric charge Q, which is proportional to the exposed surface and the size of the grains. In an unsensitised material, the passive film remains intact and the loop size is, therefore, reduced.

The samples tested are wire made of 304L AISI, used for the manufacturing process of stylets (Table 4). For the evaluation test, reference samples #B1 and #B2 and three samples from the supplier stock, subjected to heat treatments at 500 °C, 620 °C, and 750 °C, respectively, are used (Table 4).

The sample (see Table 4), cut transversely, is embedded in a resin and then polished to obtain a “mirror”. It is then cleaned with a mixture of acetone and ethanol, then rinsed with deionised water (resistivity of 18 MΩ cm). The resin is machined to fit the working electrode. The assembly of the electrodes, the electrochemical cell, and the measurement conditions comply with ASTM G108-94 (2015) [77]. The ASTM method G108-94 allows for a quantitative evaluation of AISI 304L stainless steels’ sensitivity to intergranular corrosion. The test consists of electrochemically analysing the cross-sectional area by a potential sweep ranging from +200 mV to −400 mV compared to a reference electrode SCE (saturated calomel electrode). For the evaluation of intergranular corrosion sensitivity, an additional parameter—the grain size index—must be taken into account, in accordance with ASTM standard E112-13 [78].

### 2.5. The Release of Cationsi

Is measured in artificial sweat according to standard EN 1811-2011+A1:2015 [48] and in a plasma bone environment (Fitton-Jackson and Burks-Peck). Their compositions are as follows:

- Artificial sweat according to standard EN 1811-2023-4 [79], with the following chemical composition: 1 ± 0.001 g/L of urea, 5 ± 0.001 g/L of NaCl ultra-pure, and 940 mL ± 10 µL/L of racemic lactic acid (Merck). The solution is also prepared with ultrapure quality water, conductivity (0.06–0.2 μS/cm), and without silicon (Si). The solution is filtered over a Falcon^®^ 0.22 μm sterilised membrane in order to avoid the risk of developing bacteria during the corrosion tests. The pH of the medium is 4.5.
- A plasma bone environment (Fitton-Jackson and Burks-Peck [18]) with the following chemical composition: lactic acid racemic, 460 mL/L; ultrapure NaCl: 5.3 ± 0.001 g/L; EPES: 4.8 g/L; acetic acid: 100 μL/L; α-cetoglutaric acid NaHCO_3_: 3.5 g/L; NaOH 0.2 M; with a pH of 7.5 [80].

For the extraction tests, three samples were tested for each type of milieu. Each sample, with a surface area of 9.81 cm^2^ (5 mm diameter, 60 mm length), was immersed in 10 mL of solution in a Falcon medical device tube. We tested 6 samples that underwent heat treatment at 500 °C for one hour. The scales came from the supplier stock reference B1. The sample codes are #B1A-F. The solution was prepared with ultrapure-quality water, conductivity (0.06–0.2 μS/cm), and without silicon. The extraction solutions were filtered on a 0.22 μm Falcon cellulose acetate sterilised membrane. After 168 h (± 1 h) at 37 °C, the samples were removed from the solution and were analysed by ICP OES (Inductively Coupled Plasma Optical Emission Spectroscopy) Optima 7300 V Perkin Elmer and ICP-MS (Inductively Coupled Plasma Mass Spectrometry) iCAPTM MSX Single Quadrupole Thermo Fisher. To increase the accuracy of measurement, we used a crossover technique, and the cation matrices were measured according to the following scheme:

(a) ICP-MS: Ba, Be, Cd, Co, Li, Mo, Nb, Pb, Sb, Sn, Sr, Zn, Ga, In, P; (b) ICP-OES: Al, Cr, Cu, Fe, Ni, Ti, V, Zn; (c) ICP-OES/Hydrides: As, Hg, Sb, Se.

The extraction values are expressed in μg/L and μg//cm^2^.week^−1^.

## 3. Results and Discussion

### 3.1. Uniform Corrosion

Metals and alloys immersed in an electrolytic medium generate an electrical potential that varies with time. It stabilises to a stationary value after a long immersion period. The open-circuit potential is called (Eoc) As it is primarily a surface phenomenon, this potential can vary over time because the surface nature of the electrode changes (oxidation, formation of a passive layer, or immunity).

- The open-circuit potential is a criterion for the analysis of corrosion behaviour, but it is still insufficient. The approach to the results obtained is always qualitative, but gives information on the passivity of the steel surface in our case.

The open-circuit potential is measured for all samples tested for 1 h of open-circuit immersion, which is insufficient time to obtain a stationary value. In general, the required immersion time is from 15 to 24 h. After 1 h of immersion, we reach potentiodynamic measurement stability, but the interpretation of the open-circuit potential under these conditions must be conducted with care. Consequently, we take into consideration the value of E (i = 0), which is the value of the potential of passage of the cathodic zone in the anodic zone, which is the Ecorr (Table 5). According to the results obtained (Table 5), it can be observed that the E values (i = 0) vary between −59 mV and +13 mV, without being able to show a discriminating trend in relation to the operative range of the manufacture of stylets. On the other hand, after heat treatment, the values of E (i = 0) drop sharply to values between −166 mV and −280 mV. It can be concluded that the wire is probably sensitised to intergranular corrosion.

**Figure 8:**
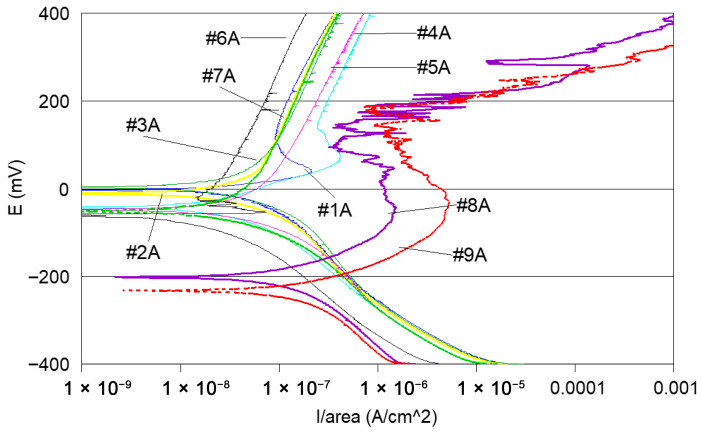
Potentiodynamic polarisation curves of series A samples.

**Figure 9:**
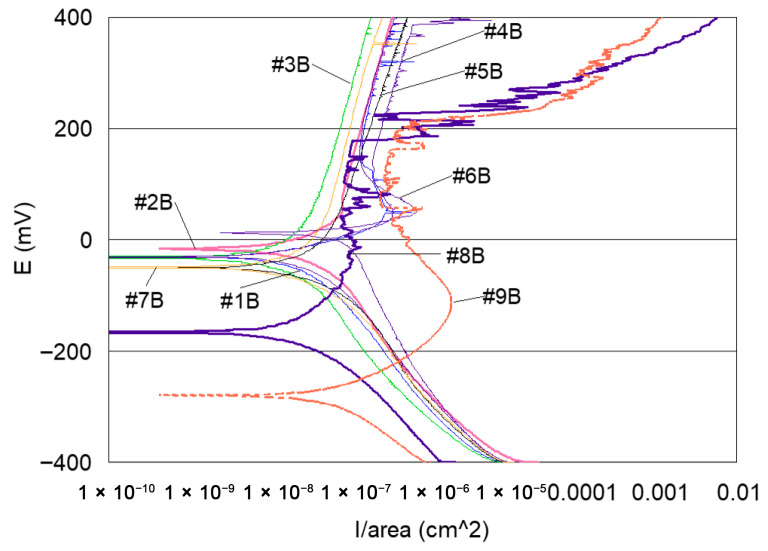
Potentiodynamic polarisation curves of series B samples.

- Potentiodynamic polarisation curves: For the series of samples studied (Table 2), potentiodynamic polarisation curves for each manufacturing step are shown in Figure 7 and Figure 8. Overall, a good reproducibility is observed. However, depending on the stage of manufacture, these polarization curves can be very different. In other words, a manufacturing step can radically change the corrosion behaviour of the wire. Thus, the analysis of polarisation curves can provide information if there is an awareness process for each step examined. To obtain a better picture of this, we group the curves by series A and by series B (Figure 8 and Figure 9).

**Figure 10:**
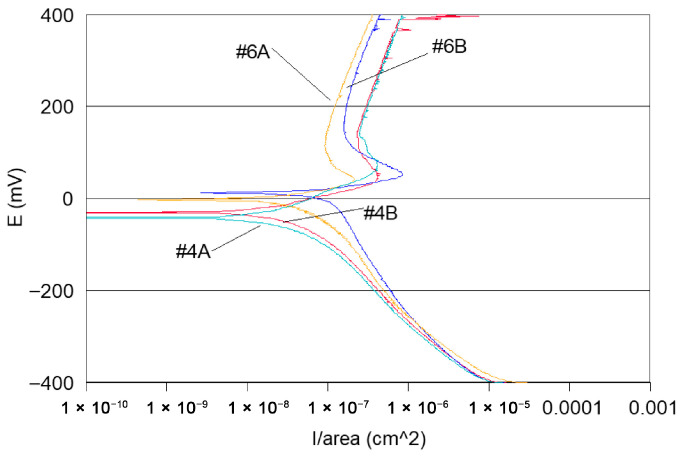
Potentiodynamic polarisation curves of series #4A, #4B and #6A, #6B samples.

**Figure 11:**
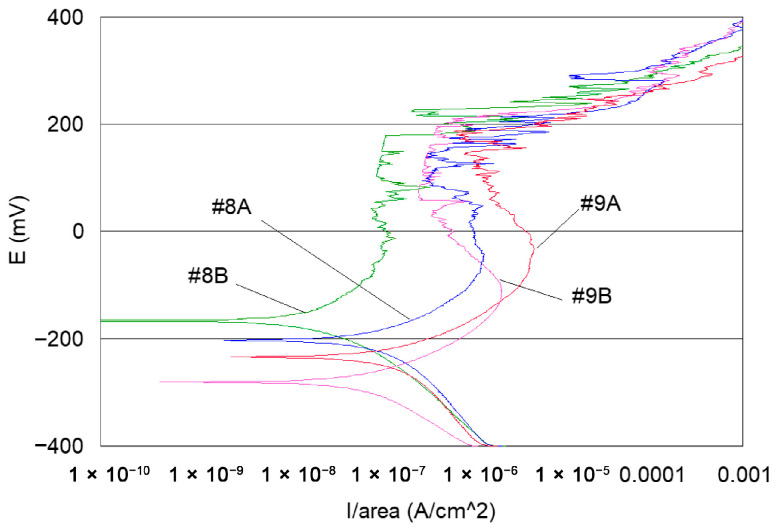
Potentiodynamic polarisation curves of series #8 A, B samples after heat treatment at 750 °C and #9 A, B samples after heat treatment at 620 °C.

If one considers all the polarisation curves shown in Figure 7 and Figure 8, several remarks can be made, as follows:

- Curves #4A, #6A and #4 B4, #6B (cleaning step) reveal a peak (Figure 10). The peaks observed in the wash steps are probably due to dissolved deposits or de-passivated–re-passivated oxide layers on the surface of the wire.
- If we examine curves #8A and #9A, compared to the other polarisation curves (Figure 10), we notice a clear difference in the behaviour of the 304L wire. The values of E (i = 0) are −202 mV and −228 mV, respectively, the anode current increases strongly from 10^−6^ to 10^−2^ A, and the curves show very important disturbances.
- In the heat treatment step, 750 °C heat treatment is applied to the styli (treatment used by the digger code #8), and for another series of the code #9, styli are treated at 620 °C, a temperature proposed by ASTM 262-15 [68] Figure 11.

It can, therefore, be said that in this stage (heat treatment), there is probably intergranular sensitisation of the yarn to corrosion. This assessment technique makes it difficult to determine the type of awareness has been experienced. Is it sensitivity to intergranular corrosion? Or another type of morphology?

It can also be observed that the polarisation curves of samples #8A, B and #9 A, B have very different appearances compared to the other polarisation curves (Figure 8, Figure 9 and Figure 10).

They show E (i = 0) values of −166 mV and −280 mV, respectively, and significant disturbances, with anodic currents of the order of a few tens of mA. The stylus is, therefore, sensitised over at least 5 cm in length (Figure 3).

- The other curves grouped together from samples A and B in Figure 8 and Figure 9 do not reveal peaks, and their anode currents are in the current zone from 10^−7^ to 10^−6^ A. It can, therefore, be assumed that in these stages of the operating ranges, there is no corrosion sensitisation.

In summary, the analysis of the polarisation curves allows us to affirm that significant awareness to the corrosion of the 304L wire is exhibited during the heat treatment step.

- *Coulometric analysis:* The surfaces under the polarisation curves are integrated and the results obtained are expressed in μC/cm^2^. In other words, the amount of current consumed for the electrochemical degradation of steel is integrated into an anodic scanning range between E (i = 0) and +400 mV ECS (Table 5) This is, therefore, a quantitative way of expressing the degree of awareness of steel in its passage through the manufacturing operating range.

Examination of the results shows a clear difference between samples that underwent heat treatment and those that did not. Samples taken from styli without heat treatment reveal a charge quantity between 35 and 183 μC/cm^2^. Heat treatment greatly increases the amount of electric charge, with integrated values between 56,000 and 33,800 μC/cm^2^.

According to Table 5, the samples tested can be grouped into the following three groups:

- A first group that underwent an operating range without heat treatment.
- A second group that underwent “heat treatment”, but is not deliverable to 620 °C.
- A third group that underwent “heat treatment” at 500 °C and is acceptable for delivery.

**Figure 12:**
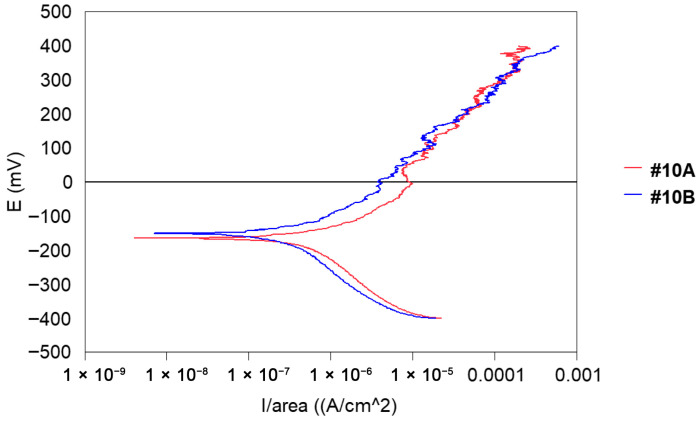
Potentiodynamic polarisation curves of series #10 A, B samples after heat treatment at 500 °C.

For the polarisation curves, measurements for series #10 are shown in Figure 12. Samples A and B were taken from a finished stylet (end of the operating range) ready for delivery. This series was considered acceptable, i.e., there was no corrosion on the surface of wires that will allow for non-delivery.

Indeed, the polarisation curves do not reveal any disturbance and the measured anode currents vary between 10^−5^ and 10^−4^ A. It will be necessary to study the possibility of taking these values as a reference for an acceptance criterion “ready to deliver”.

### 3.2. Intergranular Corrosion

- Chemical tests

Evaluation of intergranular corrosion sensitisation: The samples were taken from three coils of wire with a diameter of 0.360 mm, following the manufacturing steps shown in Table 2. The mass losses were low, with a maximum measured value of about 200 μg/cm^2^. In steps #1 to #6, no significant differences were observed between the Coil 1, Coil 2, and Coil 3 batches. No intergranular corrosion sensitisation was detected; these samples were not heat-treated (see Table 6). Scanning electron microscope observation also did not reveal intergranular corrosion. In contrast, the straightening (#3A) and heat treatment (#8A and #9A) steps resulted in mass losses. It can, therefore, be considered that a corrosion process affected the surface of the wire.

Electrochemical measurements by potentiodynamic scanning and coulometric analysis confirmed that the heat treatment sensitised the steel to corrosion. When comparing the processing temperatures, it is observed that the temperature of 620 °C (#9A) induced the highest losses. The temperature of 500 °C (#10A) seemed to be a better choice, with lower losses than those observed at 750 °C, which is currently used by the stylus manufacturer (Table 6). The results presented in Table 6 are average values of the three samples evaluated for each step of the manufacturing process. 

**Figure 13:**
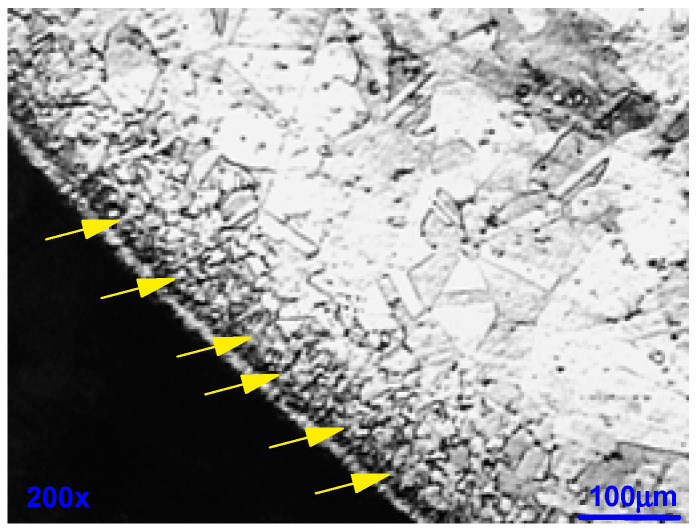
Metallographic cut sample #9A-coil 3. The outer part of the corroded wire has a structurally disturbed surface area over a depth of about 15 µm, Yellow arrows.

The metallographic cut in Figure 13 for #9A reveals that the outer part of the corroded wire has a structurally disturbed surface area over a depth of about 15 m (yellow arrows in Figure 13), at a magnification of 100x.

**Figure 14:**
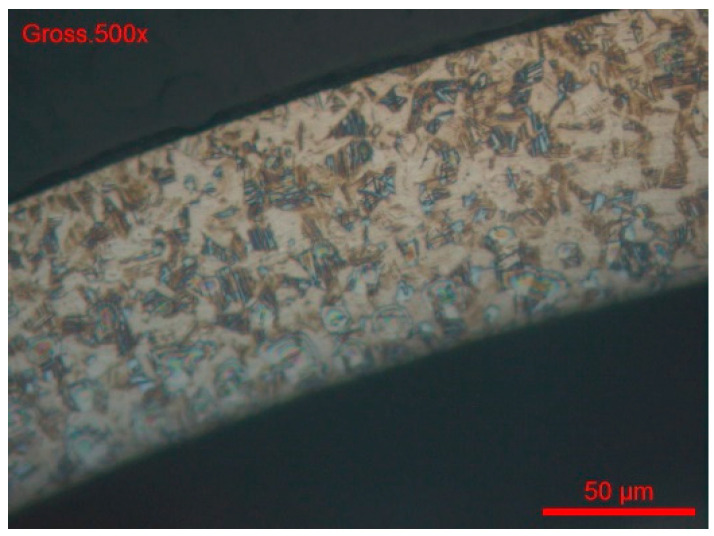
#B1-coil grain index (ASTM E112-13).

**Figure 15:**
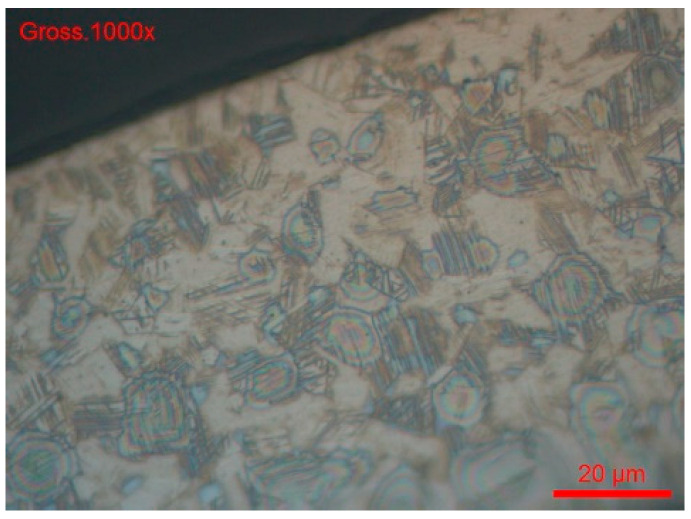
#B1-coil, magnification 1000x.

Clearly, the 24 h ASTM A262 [68] (part F) test does not reveal corrosion sensitivity of the wire, but it is difficult to say whether it is an intergranular attack (Figure 14 and Figure 15). This aspect would merit further investigation, for example, by electrochemical reactivation tests according to ASTM G108-94 [77] or a specific test for the sensitisation of 304 and 304 steels. Consequently, we studied intergranular corrosion by this method.

- Electrochemical tests: EPR method ASTM G108–94(2015) [77].

The grain index is determined according to the ASTM E112-13 method [78]. For both wire references, #B1-coil and #B2-coil, the grain index is 11 (Figure 14 and Figure 15).

According to the EPR method described in ASTM G108-94 (2015) [77], after cyclic polarisation scans, the primary evaluation parameter is the normalised charge (Pa), expressed in coulombs/cm^2^ It is calculated according to the following formula:

Pa = Q/X, where Q represents the load measured by a current integration instrument (in coulombs), normalied with respect to sample area and grain size, and X is defined by:

X = A_s_ [5.1 × 10^−3^ *e*^0.35G^], with As: sample area (in cm^2^), and G: grain size index measured at 100 magnification, according to ASTM E112-13 [78].

In the establishment of this formula, it is assumed that the charge Q results from a uniform attack on the surface of the sample, distributed exclusively along the grain boundaries, considered to have a constant width of 2 × (5 × 10^−5^) cm. It should be noted, however, that this hypothesis may not accurately reflect the actual physical mechanisms.

**Figure 16:**
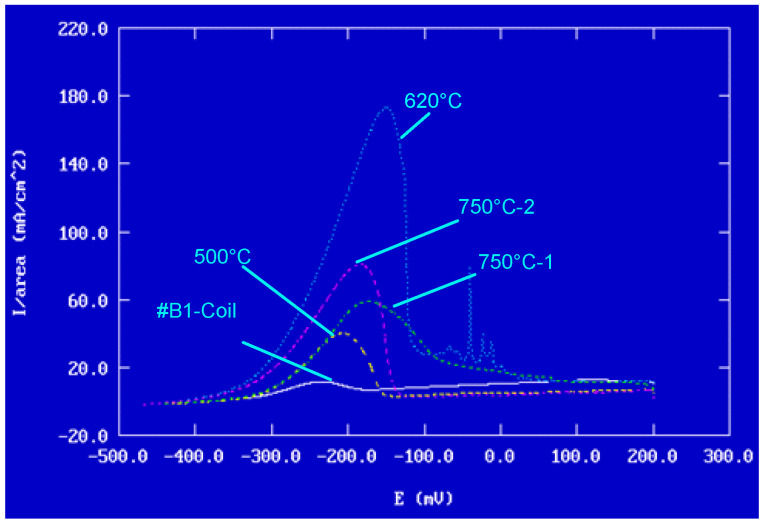
Potentiokinetic reactivation curves recorded for #B1, and heat treatment at 500 °C, 620 °C, and #750 °C.

The results of potentiokinetic electrochemical reactivation tests are presented in Table 7. Figure 16 illustrates the reactivation curves on linear axes.

In summary, eight samples from two coils (B1 and B2), according to the specifications of Table 2, were subjected to this evaluation.

The peak Ir current values, presented in Table 7, are representative of the degree of degradation induced by intergranular corrosion of the samples. A higher intensity indicates more marked degradation. Thus, as shown in Figure 16, the most pronounced sensitisation of the wires was observed after heat treatment at 620 °C, while the lowest level of sensitisation was recorded at 500 °C.

**Figure 17:**
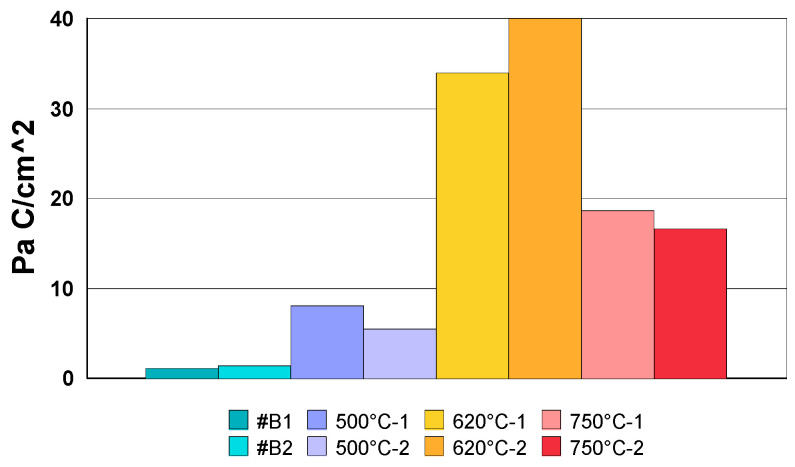
Normalised charge (Pa) measured by EPR.

The overall results (Table 7), concerning the normalised charge (Pa) calculated (see Figure 17) for all samples, clearly show that any heat treatment beyond 500 °C is not recommended for steels of type AISI 304, due to an increased risk of intergranular corrosion. Accordingly, heat treatment at 500 °C is the safest option to prioritise in the manufacturing process.

It should be noted that the evaluation method used is very sensitive. The variations observed in the results are probably because each coil has different characteristics, especially in terms of surface condition and structural changes. Considering the overall results, it appears that Coil 1 is more sensitive to intergranular corrosion

Type 304 steel is very sensitive to intergranular corrosion compared to other steels. Consequently, in the manufacturing process, great importance must be given to this type of corrosion morphology. The temperature of 620 °C is critical for generating the process and, therefore, is used in the ASTM tests (A262-15 and G108-94) for evaluating intergranular corrosion. The goal is to nearly reach the behaviour of the wire in the raw state (#B1 or #B2).

The susceptibility of stainless steels to intergranular corrosion is not always attributable to the precipitation of chromium carbides induced by heat treatment. Under certain conditions, the formation of intermetallic compounds such as (Fe,Cr)Mo or (Cr,Ni,Fe) P may also occur. 

According to Stonawská et al. [81], the structural sensitisation of 316L steel results mainly from the precipitation of secondary phases along grain boundaries. The work of Liu et al. on 316L steels [82], as well as that of Fujii et al. on 304 steel [83], also confirms this observation.

According to Liu et al. [84], chromium-depleted areas near the grain boundaries are prime sites for corrosion initiation in austenitic steels. Eliaz [60] points out that, thanks to the reduction in carbon content in 316L and 316LVM stainless steels, the phenomenon of sensitisation is now much less worrying than it was previously.

**Figure 18:**
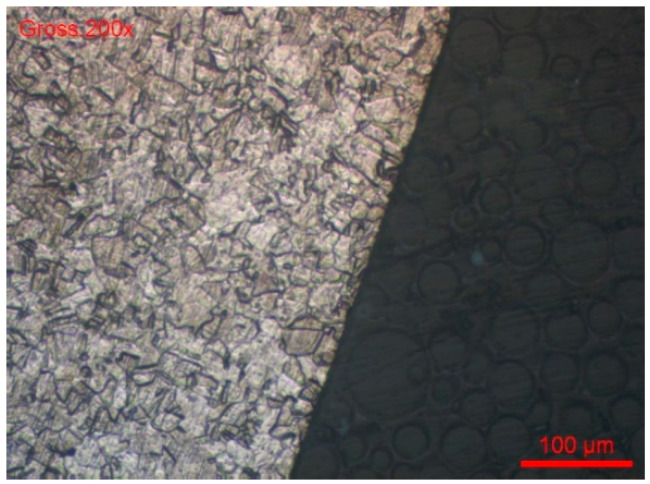
Attack used to reveal intergranular corrosion (acid oxalic 10%), 200X.

**Figure 19:**
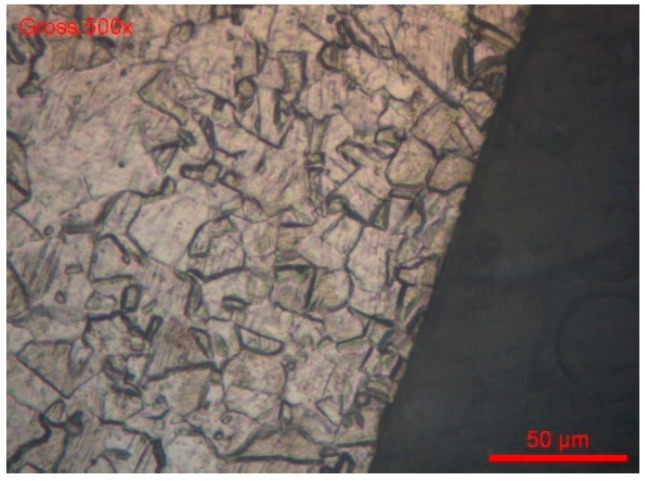
Attack used to reveal intergranular corrosion (acid oxalic 10%), 500X.

**Figure 20:**
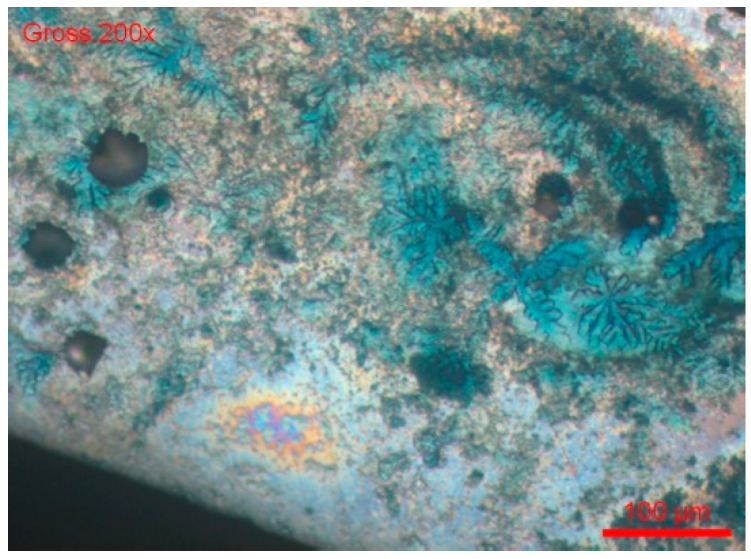
Holes and blue areas sensitive to surface corrosion (pitting crack) of steel by a ferrocyanide solution indicator.

We checked with another sample, #B1-Coil, after heat treatment at 500 °C for one hour, if the heat treatment generated an intergranular corrosion process by chemical attack in the following way:

- The sample was cross-sectionally coated and polished. The usual stainless steel attack revealed a normal structure without pronounced surface corrosion.
- We then used the ASTM A262 practice A chemical etching solution, which is used for all oxalic acid (intergranular corrosion) sensitisation tests. According to Figure 18 and Figure 19, there was no evidence of the intergranular corrosion sensitisation of steel.
- We dipped the polished sample in a solution of 9 g/L of NaCl and potassium ferrocyanide. Soon, blue spots appeared on the surface of the cut, which assumes that the steel was sensitised in a localised corrosion (pitting, crevice). All the blue zones corresponded to active zones of this form of corrosion. We can also see many pitting instances in Figure 20.

The tested sample of 304L steel does not reveal intergranular corrosion sensitisation, but it does reveal localised shape corrosion. It can be concluded that applying a 500 °C heat treatment to 304L steel grades in the stylus manufacturing process will not likely result in intergranular corrosion sensitisation.

### 3.3. The Release of Cations

Although this medical device is for single use and intended for short-term contact with living tissues, it should, nevertheless, be evaluated in accordance with ISO 10993, including cytotoxicity, sensitisation, irritation, acute systemic toxicity, subchronic toxicity, genotoxicity, hemocompatibility, and implantation. It is essential to consider the release potential of metal ions in target tissues or organs. Indeed, the release of metal cations during surgical procedures can lead to a significant toxicological risk, especially by their presence in tissues or blood circulation.

Our interest is in knowing which metal cations are released and in what quantities.

We chose the following two extraction media: artificial sweat according to EN 1811 and the Fitton-Jackson and Burks-Peck bone plasma method.

In our measurements, we did not find traces of Al < 0.5 ug/L, Cu< 2 µg/L, Mn < 0.1 µg/L, Mo < 1 µg/L, and Sr < 0.2 µg/L, so these elements do not appear in Table 2. Phosphorus and lead were not detected in the analysis of samples’ cation release.

The quantities of metal cations detected in the extraction solutions are shown in Table 8. It was observed that the concentrations released were higher in the artificial sweat medium than in bone plasma. This difference was probably due to the different chemical composition and pH of the two media: artificial sweat has an acidic pH (about 4.5), while bone plasma is neutral (pH 7.5).

Another factor influencing release kinetics is the formation of insoluble compounds, which may adhere to the metal surface or precipitate into the medium. The formation of these compounds depends on both the degree of oxidation of metals and the acidity of the medium. Thus, the chemical reactivity differed significantly between the two extraction solutions.

Low concentrations of barium were detected in both media. Barium, like strontium, is likely to be derived from calcium-associated impurities commonly found in steelmaking processes. These elements are usually removed during the refining or reflow stages of steel.

The titanium found in the artificial sweat solution appeared to come from the reagents used for its preparation, as evidenced by the similar amount observed in the control sample (blank test).

For iron, concentrations in the range of mg/L were measured in artificial sweat, while chromium was released in much lower amounts (between 34 and 45 μg/L), probably as Cr^+3^. This chromium would come from the surface of the corrosion-sensitive wire, at a depth estimated at about 15 microns (see Figure 15).

The quantities of nickel released into artificial sweat were in the order of a few hundred μg/L. Expressed in μg/cm^2^ week, they were 0.05, 0.12, and 0.10 respectively. These values comply with the limits imposed by European Union UE [53]. Piercing in the Human Body”, which specifies a maximum nickel release rate of 0.2 μg/cm^2^ per week. It should be noted that these medical devices, styli in this case, are invasive elements introduced into the human body.

Overall, the measured amounts of chromium, iron, and nickel were in the nanogram range and even lower if we consider that the actual contact time with living tissue was a few hours, not a week. Therefore, we consider it justified to state that the toxicological risks associated with contact between the device and living tissues are unlikely.

### 3.4. Nickel Release and the Manufacturing Process

In our laboratories, numerous nickel extraction tests were carried out according to EN 1811-2011+A1:2015 [48] on 304 and 316L steels produced by six different steelmakers located in the European Union, Japan, and the United States. The results revealed very significant differences in the quantities of nickel released, although the chemical composition of steels complied with current standards (AISI, DIN, and AFNOR). These variations depended largely on the steel producer.

It is important to emphasise that, even if the steels met the classification standards, their behaviour in terms of nickel release differed significantly from one manufacturer to another. These differences are explained by several parameters, such as the production processes, the casting volume, the addition elements, the deoxidising agents, etc. Some steelmakers practice steel refilling procedures.

Heat treatments generally reduced nickel release rates, while the surface state of materials had little influence. On the other hand, curing processes had a significant impact: an increase in hardness significantly reduced corrosion resistance, which led to a noticeable increase in the amount of nickel released (see Table 9).

Another determining factor was the presence of inclusions and secondary phases in the microstructure of steels, which promote nickel release. Aware of these issues, subcontractors impose very strict specifications on steelmakers regarding the manufacturing conditions for steels intended for sensitive applications.

## 4. Conclusions

Styli are invasive medical devices used during short-term procedures in the human body. Their manufacturing presents two major challenges, as follows:

- The first difficulty concerns respecting a manufacturing range with very rigorous parameters, in order to respect narrow tolerances and passivation processes to guarantee the chemical inertia of the device surface.
- The second difficulty, even more complex, lies in the heat treatments applied to stainless steel. Indeed, a poor mastery of these treatments can induce an awareness of intergranular corrosion, a phenomenon that compromises the resistance of the material and makes the medical device unusable.
- It is also important to be aware of the biological risks associated with using these materials. During a surgical procedure, the device may release metal cations such as chromium (Cr), nickel (Ni), molybdenum (Mo), manganese (Mn), iron (Fe), and traces of other chemical elements into a patient’s tissues, blood, or plasma. These rejections can have toxic consequences or cause inflammatory reactions.
- The originality of this study lies in its integrated and systematic approach, which scientifically establishes the link between structural sensitisation induced by thermal treatments, electrochemical behaviour, cleaning processes, and surface passivation and the different stages of the industrial process for manufacturing medical devices made with stainless steel 304L. This work brings concrete elements to optimise the manufacturing parameters, in order to guarantee durable resistance to corrosion and the optimal clinical safety.

## Figures and Tables

**Table 1:** Chemical composition of the grades of austenitic steels used in corrosion tests.

Code	DIN	AISI	C	Si	Mn	P	S	Cr	Mo	Ni	Other
#1	1.4306	304L	<0.030	<1.50	<1.50	<0.035	<0.020	17.0–20.0	--	8.0–12.0	N 0.10-0-20

**Table 2:** Sampling.

Samples	Manufacturing Process Steps
#1A	Coil delivered ^1^
#1B	
#2A	Mechanical straightening ^2^
#2B	
#3A	Wire drawing ^3^
#3B	
#4A	Cleaning ^4^
#4B	
#5A	Bevelled, rounded ^5^
#5B	
#6A	Cleaning ^6^
#6B	
#7A	Cold forming ^7^
#7B	
#8A	Heat treatment ^8^ at 750 °C
#8B	
#9A	Heat treatment at 620 °C
#9B	
#10A	Heet treatment at 500 °C
#10B	

**Table 3:** ASTM A262-15 [68] standard practice for detecting susceptibility to intergranular corrosion in austenitic stainless steels.

Designation	Test	Temperature	Testing Time	Applicability	Evaluation Method
Practice E	6% CuSO_4_ 16% H_2_SO_4_ with Cu metallic	Boiling	24 h	Chromium Carbide	Examination for fissures after bending
Practice F	CuSO_4_ 50% H_2_SO_4_ with Cu metallic	Boiling 125 °C	120 h	Chromium Carbide in 316 and 316L	Weight loss/ Corrosion rate

**Table 4:** Test samples used in investigation of intergranular 304L behaviour.

Code	Description
#B1	Wire ϕ 5 mm diameter from supplier stock (reference)
#500_1	Wire ϕ 5 mm supplier +500 °C heat treatment, 1 h
#620_1	Wire ϕ 5 mm supplier stock +620 °C heat treatment, 1 h
#750_1	Wire ϕ 5 mm supplier stock +750 °C heat treatment, 1 h
#B2	Wier ϕ 5 mm from supplier stock (second reference)
#500_2	Wire ϕ 5 mm supplier +500 °C heat treatment, 1 h
#620_2	Wire ϕ 5 mm supplier stock +620 °C heat treatment, 1 h
#750_2	Wire ϕ 5 mm supplier stock +750 °C heat treatment, 1 h

**Table 5:** Coulometric analysis.

Code	E(i = 0)	Q E(i = 0) à 400 mV	Remarques
	mV	µC/cm^2^	Process Steps
#1A	−2	66	Coil delivered
#1B	−30	104	
#2A	−10	64.	Mechanical straightening
#2B	−14	75	
#3A	−4.9	70	Wire drawing
#3B	−29	40	
#4A	−41	163	Cleaning
#4B	−26	180	
#5A	−47	117	Bevelled, Rounded
#5B	−49	104	
#6A	−59	35	Cleaning
#6B	+13	112	
#7A	−52	69	Cold forming
#7B	−48	56	
#8A	−202	56,180	Heat treatment at 750 °C
#8B	−166	196,600	
#9A	−228	339,800	Heat treatment at 620 °C
#9B	−280	154,600	
#10A	−165	29,460	Heat treatment at 500 °C
#10B	−151	37,320	

**Table 6:** Mass loss after intergranular corrosion sensitivity test.

Stage		Initial	Final	Perte	Surface	Perte
		m_1_ [g]	m_2_ [g]	Δm [μg]	[cm^2^]	Δm [μg.cm^−2^]
#1A	Lot 1	0.0161	0.0161	0	0.46	0
	Lot 2	0.01509	0.01509	0	0.43	0
	Lot 3	0.01608	0.01608	0	0.46	0
#2A	Lot 1	0.02399	0.02396	30	0.68	44
	Lot 2	0.02363	0.02363	0	0.67	0
	Lot 3	0.02288	0.02287	10	0.65	15
#3A	Lot 1	0.01696	0.01686	100	0.48	208
	Lot 2	0.01865	0.01856	90	0.53	170
	Lot 3	0.01465	0.01463	20	0.42	48
#5A	Lot 1	0.03132	0.03127	50	0.89	56
	Lot 2	0.03323	0.03322	10	0.94	11
	Lot 3	0.03271	0.03266	50	0.93	54
#6A	Lot 1	0.02281	0.02272	90	0.65	139
	Lot 2	0.02363	0.02363	0	0.67	0
	Lot 3	0.02389	0.02389	0	0.68	0
#8A	Lot 1	0.01486	0.01482	40	0.42	95
	Lot 2	0.02520	0.02516	40	0.72	56
	Lot 3	0.03154	0.03149	50	0.90	56
#9A	Lot 1	0.01482	0.01474	82	0.64	128
	Lot 2	0.02307	0.02299	75	0.65	115
	Lot 3	0.03264	0.03256	79	0.65	122
#10A	Lot 1	0.01383	0.01382	10	0.39	25
	Lot 2	0.02394	0.02392	20	0.68	29
	Lot 3	0.03095	0.0309	50	0.88	57

**Table 7:** Potentiokinetic electrochemical reactivation results.

Code	E_oc_ [mV]	I_r_ [mA/cm^2^]	Q [C/cm^2^]	P_a_ [C/cm^2^]
#B1-Coil	−387	11.84	0.28	1.15
#B2-Coil	−410	8.21	0.33	1.37
#500_1	−388	59.22	1.94	8.10
#500_2	−410	30.70	1.33	5.54
#620_1	−404	173.30	8.17	34.04
#620_2	−395	162.20	9.94	41.42
#750_1	−407	71.86	4.49	18.69
#750_2	−407	59.22	4.01	16.70

E_oc_ = Initial open circuit potential, I_r_ = maximum anodic current density.

**Table 8:** Metal cations are released in artificial sweat and plasma bonne.

Milieu	Code	Ba	Cr		Fe		Ni		Ti
		µg/L	µg/L	µg/cm^2^ Week	µg/L	µg/cm^2^ Week	µg/L	µg/cm^2^ Week	µg/L
	Blank	<0.2	<1		3.8		<1		1.8
Sweat	#B1-A	1.3	33.6	0.034	1325	1.35	45.3	0. 05	2.0
	#B1-B	1.6	36.0	0.037	2262	2.31	115.3	0.12	2.2
	##B1-C	1.5	45.2	0.046	1475	1.51	101.8	0.10	2.5
Plasma bone	Blank	0.3	<1		1.5		<1		<0.5
	#B1-D	0.8	<2		58.8	0.06	22.5	0.02	<0.5
	#B1-E	0.9	2.6	0.03	17.4	0.07	26.8	0.03	<0.5
	#B1-F	0.9	2.0	0.02	46.8	0.05	33.4	0.04	<0.5

**Table 9:** Factors influencing the amounts of nickel released during the manufacturing process.

	Parameters	Quantity of Ni Release
Raw Materials	Variable, In Function of the Lot	Strong Dispersion
Heat Treatment	100% N_2_ 100% H_2_	Strong Decrease Light Decrease
Surface	Rough Polished Satiny	Slight Influence
Work Hardening	Strain >10%	Increase
Structure	Inclusions and Second Phases	Increase

## Data Availability

The original contributions presented in this study are included in the article. Further inquiries can be directed to the corresponding author.
